# Time trends in physical activity in the Tromsø study: An update

**DOI:** 10.1371/journal.pone.0231581

**Published:** 2020-04-14

**Authors:** Bente Morseth, Laila Arnesdatter Hopstock

**Affiliations:** 1 School of Sport Sciences, Faculty of Health Sciences, UiT The Arctic University of Norway, Tromsø, Norway; 2 Department of Community Medicine, Faculty of Health Sciences, UiT The Arctic University of Norway, Tromsø, Norway; Linneaus University, SWEDEN

## Abstract

Although the health benefits of physical activity are well documented, a large proportion of the population fails to meet current guidelines for physical activity. In order to develop evidence-based public health policies, surveillance of physical activity prevalence and trends is essential. The main aim of this study was to present updated data on physical activity trends in a Norwegian general population over the last decades. Data were collected from 40 690 individuals (50% men) aged ≥20 years participating in at least one of six surveys of the population-based Tromsø Study between 1979 and 2016. Age-standardized prevalences and trends in leisure-time and occupational physical activity were obtained from three questionnaires used in the different surveys. We observed an increase in the proportion engaging in exercise in leisure-time between 1994–95 and 2001 (*p* <0.001). Based on a different questionnaire, the age-standardized prevalence of engagement in exercise in leisure-time increased significantly from 16% in 2001 to 23% in 2007–08, and further to 28% in 2015–16 (*p* <0.001). The proportion who reported exercising approximately every day increased from 19% in 2007–08 to 28% in 2015–16 (*p* <0.001). The age-standardized prevalence of sedentary occupational activity increased from 53% in 2007–08 to 57% in 2015–16 (*p* <0.001), which extends the gradual increase from 36% in 1979-80.The present study extends previous findings from the Tromsø Study by demonstrating an increase in the proportion exercising regularly over the last three decades. This increase may partially counteract the gradual increase in the proportion with sedentary occupational activity.

## Introduction

Although the health benefits of physical activity are well documented [1], a large proportion of the population fails to meet current guidelines for physical activity [2]. According to a large study including data on 1.9 million participants from 168 countries, the global age-standardized prevalence of insufficient physical activity was 27.5% in 2016 [2]. In order to develop evidence-based public health policies, surveillance of physical activity prevalence and trends is essential.

Time trends in physical activity are documented in repeated cohort studies, of which most are conducted during a period of up to 20 years between 1980 and 2010 [3–11]. Previous studies consistently show that occupational physical activity levels gradually decline over time [3, 4, 10–13], whereas leisure-time physical activity seems to have increased over the last decades [3–11]. A study from Finland shows an increase in participants engaging in high leisure-time physical activity levels between 1982 and 2012, from 21% to 33% in men and 12% to 27% in women [4]. An increase in leisure-time physical activity level has also been reported in the UK [10], Spain [5], Sweden [7], Canada [8], US [14], and Denmark [9]. In Norway, previous studies of physical activity trends show ambiguous direction and patterns [15–17].

In a previous paper, we described secular trends in leisure-time and occupational physical activity between 1979 and 2008 in the population-based Tromsø Study [18]. The aim of this study was to present updated trends in leisure-time and occupational physical activity by adding previously unpublished data on physical activity from the Tromsø Study.

## Methods

### Study population

The Tromsø Study is a community-based cohort study with repeated surveys conducted in the municipality of Tromsø, Norway [19]. The study was initiated in 1974 (Tromsø 1), with repeated health surveys in 1979–80 (Tromsø 2), 1986–87 (Tromsø 3), 1994–95 (Tromsø 4), 2001 (Tromsø 5), 2007–08 (Tromsø 6) and 2015–16 (Tromsø 7). The first survey comprised men only; thereafter both women and men were invited. Response rates ranged from 65 to 79% (Tromsø 1: 83%; Tromsø 2: 85%; Tromsø 3: 81%; Tromsø 4: 77%; Tromsø 5: 79%; Tromsø 6: 66%; and Tromsø 7: 65%) [19]. The invited samples varied from large random samples to total birth cohorts or all adult inhabitants in Tromsø municipality [19].

**Fig 1:**
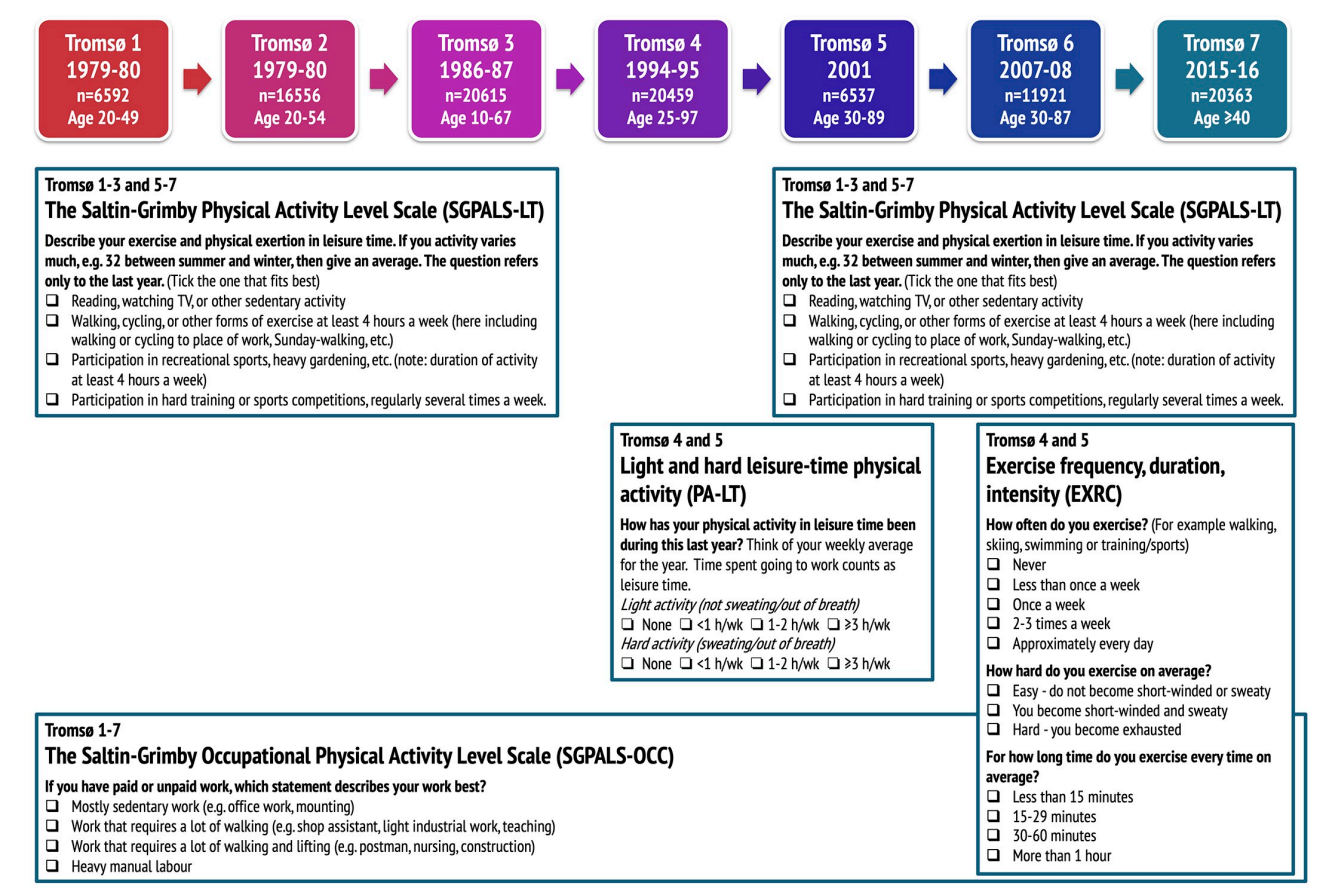
Self-reported measurement of physical activity in the Tromsø Study 1974–2016 (Tromsø 1–7). Numbers refer to participants with valid data on self-reported physical activity.

Eligible for this study were participants in Tromsø 2–7 who answered questions about leisure-time and occupational physical activity in one or more surveys (Fig 1). Altogether 37 193 participants with valid data on leisure-time physical activity and 40 690 participants with valid data on occupational physical activity contributed to the analyses. We report age-standardized prevalences of physical activity in women and men using 222 852 observations for leisure-time physical activity and 86 799 observations for occupational physical activity.

### Ethics approval

The Tromsø Study was approved by the *Regional Norwegian Data Protection Authority* and the *Regional Committee of Medical and Health Research Ethics* in Norway (REC North), and conducted according to the principles expressed in the Declaration of Helsinki. All participants in Tromsø 4─7 gave written informed consent.

### Physical activity measurements in the Tromsø Study

In all seven surveys of the Tromsø Study, physical activity was measured using questionnaires (Fig 1). Three different physical activity questionnaires were used;

1. The Saltin-Grimby Physical Activity Level Scale (SGPALS) [20, 21] measures leisure-time and occupational physical activity separately (referred to as *SGPALS-LT* and *SGPALS-OCC*, respectively). The SGPALS-LT was used in Tromsø 1, 2, 3, 5 (only in participants aged <70 years), 6, and 7, whereas the SGPALS-OCC was used in all surveys (Tromsø 1–7).
2. A questionnaire on leisure-time physical activity (referred to as *PA-LT*), which was used in Tromsø 4 and 5 [22];
3. An exercise questionnaire [23] (referred to as *EXRC*), used in Tromsø 6 and 7.

#### The SGPALS-LT questionnaire

In the SGPALS-LT, the participants were asked to tick on of the following alternatives: 1) *“Reading*, *watching TV*, *or other sedentary activity”* (Inactive), 2) “*Walking*, *cycling*, *or other forms of exercise at least four hours a week”* (Light physical activity), 3) *“Participation in recreational sports*, *heavy gardening etc*. *at least four hours a week”* (Moderate physical activity), or 4) *“Participation in hard training or sports competitions regularly several times a week”* (Vigorous physical activity) (Fig 1). The two highest levels of physical activity (categories 3 and 4) were combined and referred to as exercise in this paper.

The SGPALS-LT [20, 21] has been thoroughly validated [24] and shown to be positively correlated with accelerometry-measured physical activity [24], maximal oxygen uptake [24, 25], and energy expenditure measured by the doubly labelled water method [26] in a dose-response relationship, ensuring adequate concurrent validity of the SGPALS-LT. The predictive validity of the SGPALS-LT has been shown for several disease outcomes such as cardiovascular disease [16, 27], atrial fibrillation [28], and fracture [29] risk. A study of the reproducibility of the SGPALS-LT showed 86% agreement between assessments one month apart [30].

#### The PA-LT questionnaire

The PA-LT questionnaire asks for hours per week in light and hard physical activity in leisure-time over the last year [22] (Fig 1). Both the light and hard physical activity questions have four response alternatives: None; <1 hour/week; 1–2 hours/week; ≥3 hours/week (Fig 1).

The PA-LT questionnaire has been scarcely validated, showing non-significant correlations between light physical activity and maximal oxygen uptake or energy expenditure [31]. The hard physical activity question showed a correlation of 0.46 with maximal oxygen uptake and non-significant correlations with accelerometry-measured physical activity [31].

#### The EXRC questionnaire

The EXRC questionnaire includes three questions on exercise intensity, frequency and duration, respectively [23]. The response options are shown in Fig 1.

The EXRC questionnaire has been used in a few studies and scarcely validated, showing a dose-relationship between maximal oxygen uptake and exercise intensity, frequency, and duration, and high reproducibility [32].

#### The SGPALS-OCC questionnaire

The Saltin-Grimby Occupational Physical Activity Level Scale [20, 21] (SGPALS-OCC) relates to both paid and unpaid work and asks the participants to tick on of the following alternatives: 1) “*Mostly sedentary work*”, 2) “*Walking*” *(work requiring a lot of walking)*, 3) “*Walking and lifting*” *(work requiring a lot of walking and lifting)*, or 4) “*Heavy manual labour*”.

The SGPALS-OCC has shown low concurrent validity with no association between SGPALS-OCC and maximal oxygen uptake [24, 31], and varying predictive validity [28, 33].

### Statistical analyses

We report age-standardized prevalences of physical activity according to the SGPALS-LT, overall and in 10-year age-groups, between 2007–08 (Tromsø 6) and 2015–16 (Tromsø 7) and how these prevalences extend previously published trends from 1979–80 (Tromsø 2) [18]. Additionally, we report changes in self-reported light and hard physical activity based on the PA-LT questionnaire between 1994–95 (Tromsø 4) and 2001 (Tromsø 5). Furthermore, we describe changes in exercise intensity, duration, and frequency from the EXRC questionnaire between 2007–08 (Tromsø 6) and 2015–16 (Tromsø 7). Finally, we show the development in occupational physical activity according to the SGPALS-OCC between 1979–80 (Tromsø 2) and 2015–16 (Tromsø 7). Age-adjusted prevalences of physical activity were estimated using direct standardization. Changes in prevalence of physical activity between each survey and linear trends across surveys were tested using generalized estimating equations (GEE) analyses. Although each survey was treated as a cross-sectional cohort, some individuals attended more than one survey; therefore, the GEE method was indicated in order to account for dependency of observations. When visual inspection of the data indicated non-linear associations, a polynomial term (squared) was added to the model to fit curves. Two-sided *p* values <0.05 were considered statistically significant. All analyses were performed using IBM SPSS Statistics, version 25 (IBM Corporation, Armonk, NY, USA).

## Results

### Trends in exercise participation 1994–2001, according to the PA-LT questionnaire

**Fig 2:**
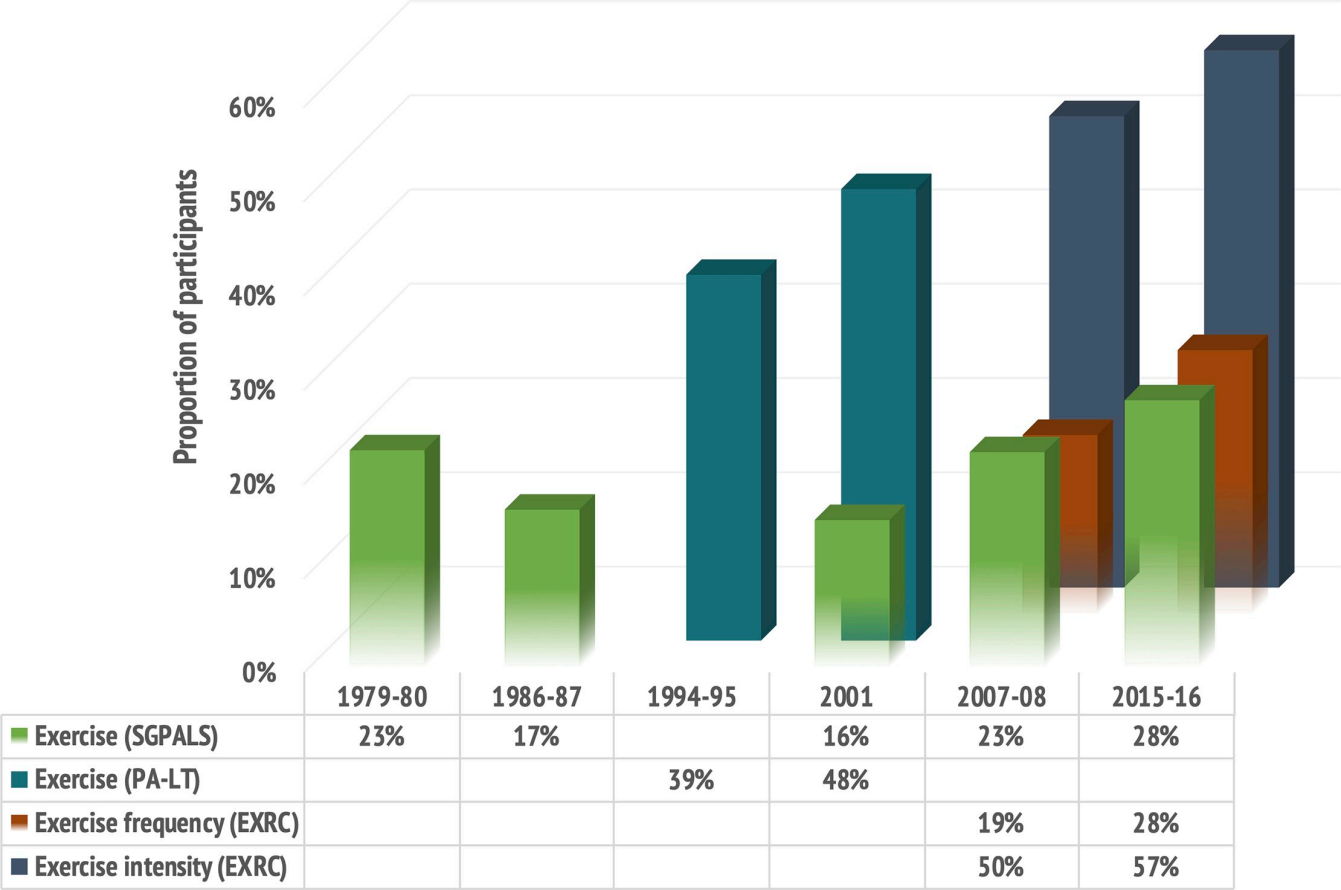
Trends in the proportion of participants engaging in exercise in leisure-time. **The Tromsø Study 1979–2016.** The bars represent the proportion of participants reporting to exercise in leisure-time, derived from four different questions about physical activity in the various Tromsø Study surveys.

Participation in exercise measured by the PA-LT questionnaire increased from 1994–95 to 2001, as the proportion of participants who reported undertaking hard physical activity more than 3 hours per week increased significantly from 1994–95 to 2001 (39% vs. 48%) (*p* <0.001) (Fig 2).

### Trends in exercise volume 2007–2016, according to the SGPALS-LT

**Fig 3:**
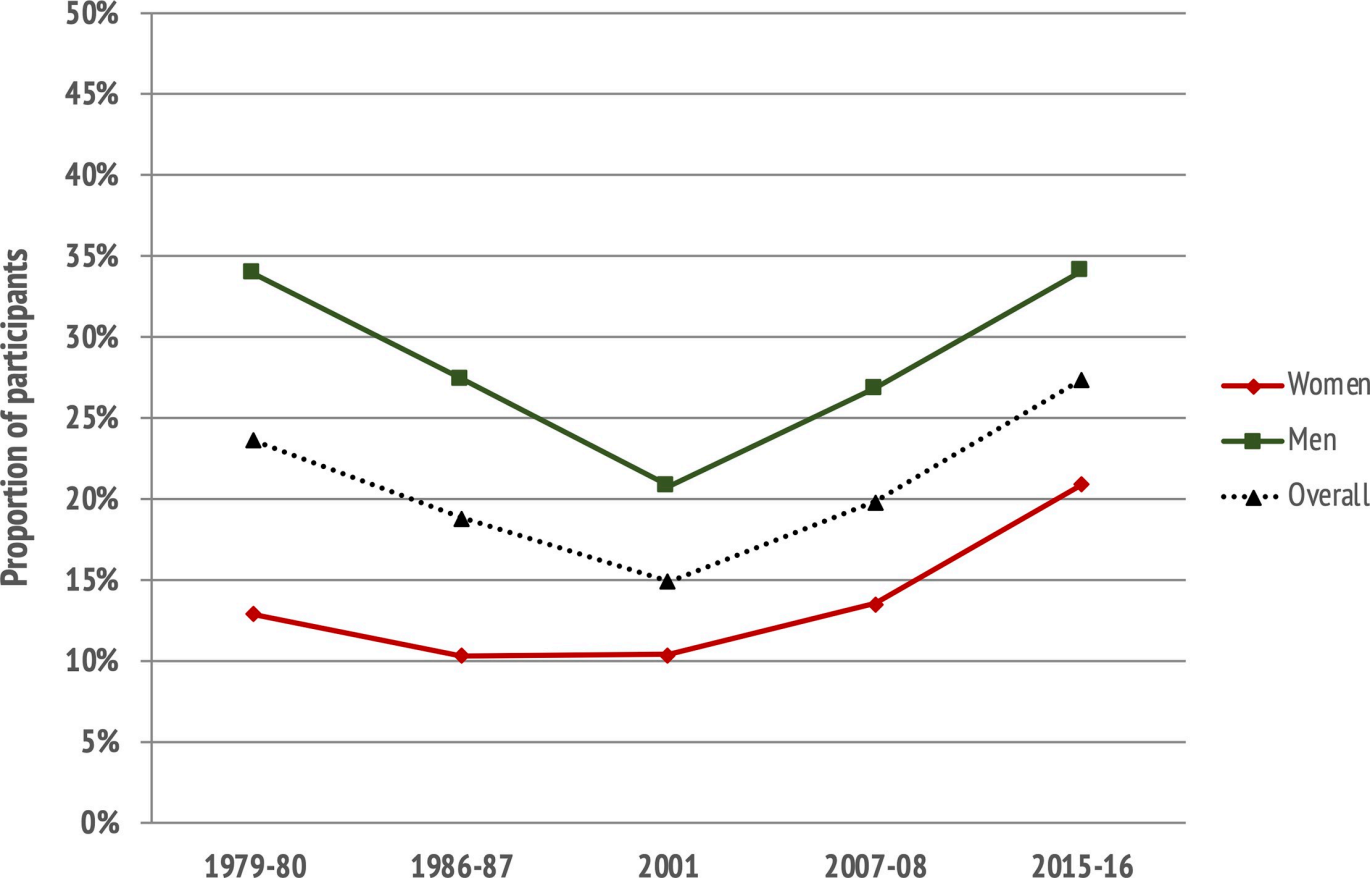
Trends in the proportion of participants engaging in exercise in leisure-time, by sex. **The Tromsø Study 1979–2016.** Prevalences are shown as separate lines for men, women, and overall. Data from 1979–80 to 2007–08 are published previously [18].

**Fig 4:**
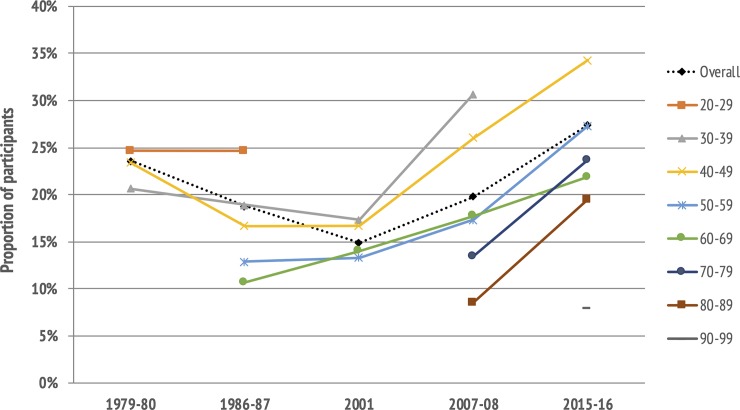
Trends in the proportion of participants engaging in exercise in leisure-time, by age. **The Tromsø Study 1979–2016.** Prevalences are shown as separate lines for age groups. Some age groups were not represented in all surveys. Data from 1979–80 to 2007–08 are published previously [18].

According to the SGPALS-LT, there was a significant increase in age-standardized prevalence of participants engaging in exercise from 23% in 2007–08 to 28% in 2015–16 (*p* <0.001), reinforcing the U-shaped trend in the proportion reporting to exercise over time (Fig 2). The trends were similar in women and men (Fig 3), and between age-groups, as the proportion engaging in exercise increased by nearly 10 percentage points in all age-groups from 2007–08 to 2015–16 (Fig 4). The age-standardized proportion of participants being inactive in leisure-time decreased from 20% in 2007–08 to 15% in 2015–16 (*p* <0.001), after remaining stable around 20% from 1979 to 2008.

### Trends in exercise frequency, intensity, and duration 2007–2016, according to the EXRC

An increasing proportion of participants reported exercising approximately every day, from 19% in 2007–08 to 28% in 2015–16 (*p* <0.001) (Fig 2). Among these, an increased proportion reported to reach an exercise intensity where they felt short winded and sweaty (50% in 2007–08 vs. 57% in 2015–16) (*p* <0.001), whereas the proportion reporting exercising more than 30 minutes per session decreased from 83% to 79% (*p* >0.001).

### Trends in occupational physical activity 1979–2008, according to the SGPALS-OCC

**Fig 5:**
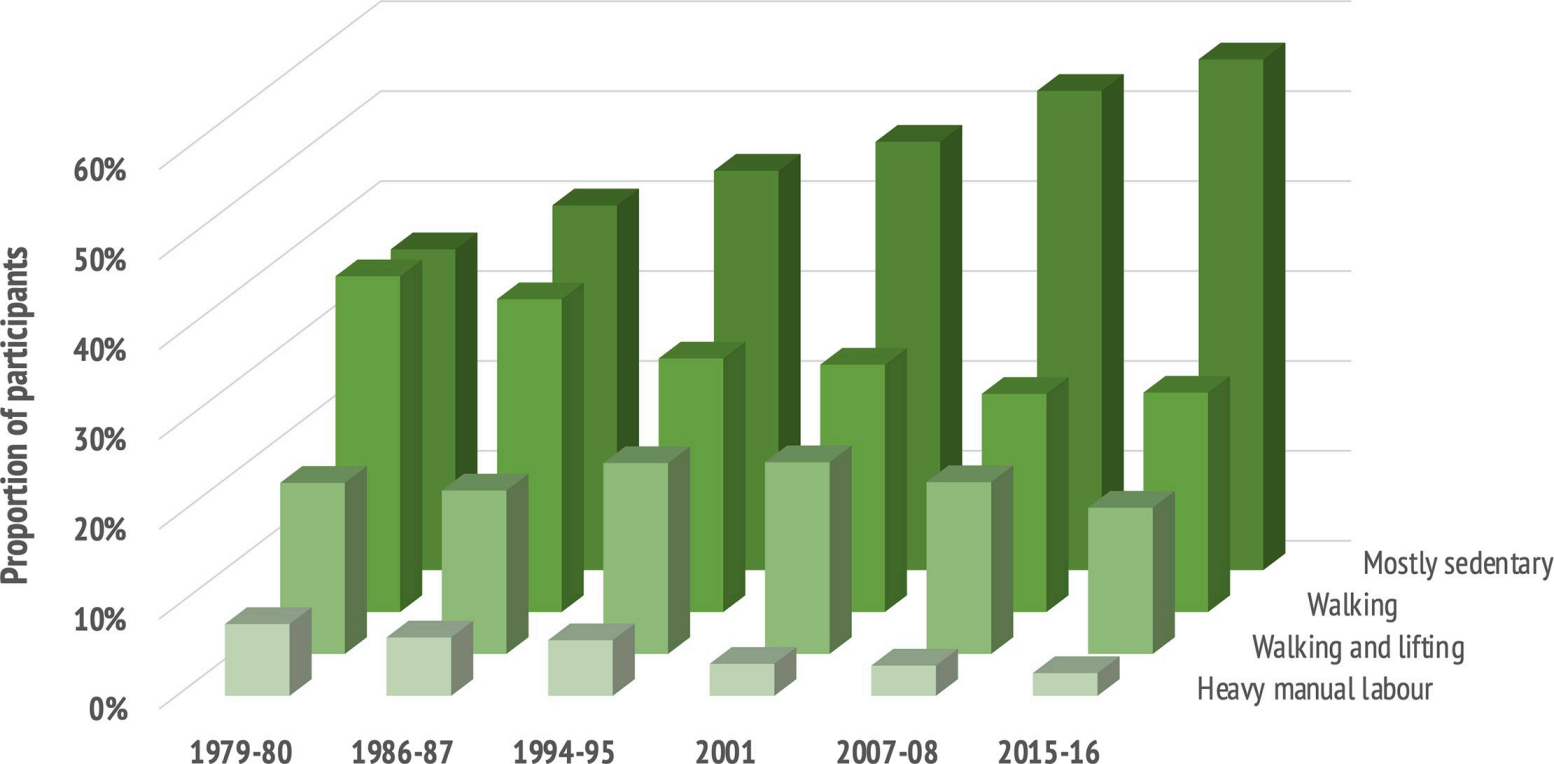
Trends in occupational physical activity 1979–2016. **The Tromsø Study.** Data from 1979–80 to 2007–08 are published previously [18].

The age-standardized proportion of participants reporting sedentary occupational activity in the SGPALS-OCC increased from 35% in 1979–80, to 40% in 1986–87, further to 45% in 1994–95, 47% in 2001, 53% in 2007–08, and recently 57% in 2015–16 (*p* <0.001) (Fig 5). This was paralleled by a decrease in walking and lifting and heavy manual labour.

## Discussion

In this cohort of Norwegian adult men and women, we observed an increasing trend of participants reporting to exercise in leisure-time over the last three decades. This increase seems to be driven particularly by an increase in exercise intensity and frequency. The increase was observed in all age groups, including ≥80 years, and in women and men. Increased exercise in leisure-time may partially counteract the gradual increase in the proportion reporting sedentary occupational activity between 1979 and 2008 [18], which we here show to be further extended to 2015–16.

The proportion of participants reporting to engage in exercise seems to depend largely on the nature of the questionnaire. The SGPALS-LT shows that 23–28% of the participants report to exercise regularly, which is relatively similar to the EXRC questionnaire with 19–28% of the participants reporting to exercise approximately every day. The PA-LT questionnaire shows somewhat higher prevalences of 39–48%. The nature of the questionnaires, with crude and discretionary categorization of physical activity levels, complicates comparison with other studies. However, if the reliability is high, as shown to be the case for the some but not all of the questionnaires used in this study [30, 31], repeated questionnaires may be valid for trend analyses.

The observed increase in participation in exercise over the last two decades is in accordance with a number of previous studies [5, 7–10], for example a Danish study showing a 6% increase in prevalence of MVPA from 1987 to 2005 [9]. However, existing research is not totally consistent, with some studies showing declining trends in leisure-time physical activity [34, 35]. Most studies have assessed the overall increase in the proportion of the population reporting engaging in leisure-time physical activity, without considering changes in physical activity intensity. Our findings suggest that the increase in exercise engagement is mainly driven by an increase in intensity and frequency, more than exercise duration.

Interestingly, we observed that the increase in participation in exercise from 2007–08 to 2015–16 is also present in the oldest age groups ≥80 years and the increase is of the same magnitude as in ages 40–79 years. This is in accordance with other studies of long-term physical activity trends that included older individuals, for example a study from Australia showing an increase in physical activity between 1998 and 2005 among individuals ≥75 years [36]. Similarly, a study from Spain demonstrated a substantial increase in leisure-time physical activity over time in women and men aged 65–79 and ≥80 years [6].

Recent findings of 6-year trends in accelerometry-measured physical activity suggest that changes in physical activity levels may be sex- and age-dependent [37]. We have previously shown that trends in leisure-time and occupational physical activity followed a similar pattern for women and men [18], although there were sex differences in prevalences; a higher percentage of men engaged in exercise [18]. This trend was further extended from Tromsø 6 (2007–08) to Tromsø 7 (2015–16).

Our findings are in accordance with previous studies on physical activity showing a decrease in occupational physical activity levels [3, 4, 10–13]. Given that people spend a lot of their time at work and only a small part of the day engaging in leisure-time physical activity, this may have a large impact of the overall physical activity level. However, the health effects of occupational physical activity are not evident [38], and a recent meta-analysis including 193 696 participants showed that men with high occupational physical activity level had an 18% increased mortality risk compared with men with low occupational physical activity, although this increased risk was not observed in women [39].

### Limitations and strengths

Physical activity has predominantly been measured by questionnaires, although recently, measurement devices such as accelerometers have been introduced. The SGPALS [20, 21] is commonly used in population studies [17, 40–43] and shows adequate concurrent and predictive validity [20]. However, overestimation of true physical activity levels is evident when participants self-report their activity level [24], indicating that the self-reported physical activity levels in this study should not be perceived as exact prevalences.

Moreover, we cannot rule out the possibility that attitude and awareness of the health benefits of physical activity may have changed over time, which may affect the participants’ perception of the questions. Furthermore, the occupational physical activity question was directed at participants with paid or unpaid work, and may have been answered by participants who were retired, unemployed, receiving disability pension, housekeepers, or students, which could potentially introduce bias.

A major strength of this study is the large sample, and high participation rates ensure high representability, which is essential in prevalence studies. Still, non-participants may differ from participants, introducing potential selection bias. Previous studies have shown that non-participants tend to have lower socioeconomic status, and higher risk of cardiovascular diseases and mortality [44]. A study of responders and non-responders in Tromsø 2 showed that the responders were more likely to be married, non-smokers, and have more sedentary work, but they did not differ regarding leisure-time physical activity levels [45].

### Conclusion

The present study shows an increase in self-reported leisure-time exercise over the last 25 years in a large, population-based cohort of adult women and men. This increase in exercise in leisure-time is important considering the continuing increase in the proportion of reported sedentary occupation.
