# Correction: Time trends in physical activity in the Tromsø study: An update

**DOI:** 10.1371/journal.pone.0242998

**Published:** 2020-11-20

**Authors:** Bente Morseth, Laila Arnesdatter Hopstock

In Fig 1, the year of Tromsø 1 should be 1974 instead of 1979–80. In addition, the Exercise frequency, duration, intensity (EXRC) questionnaire was used in Tromsø 6 and 7, not Tromsø 4 and 5. Please see the correct Fig 1 here.

**Fig 1:**
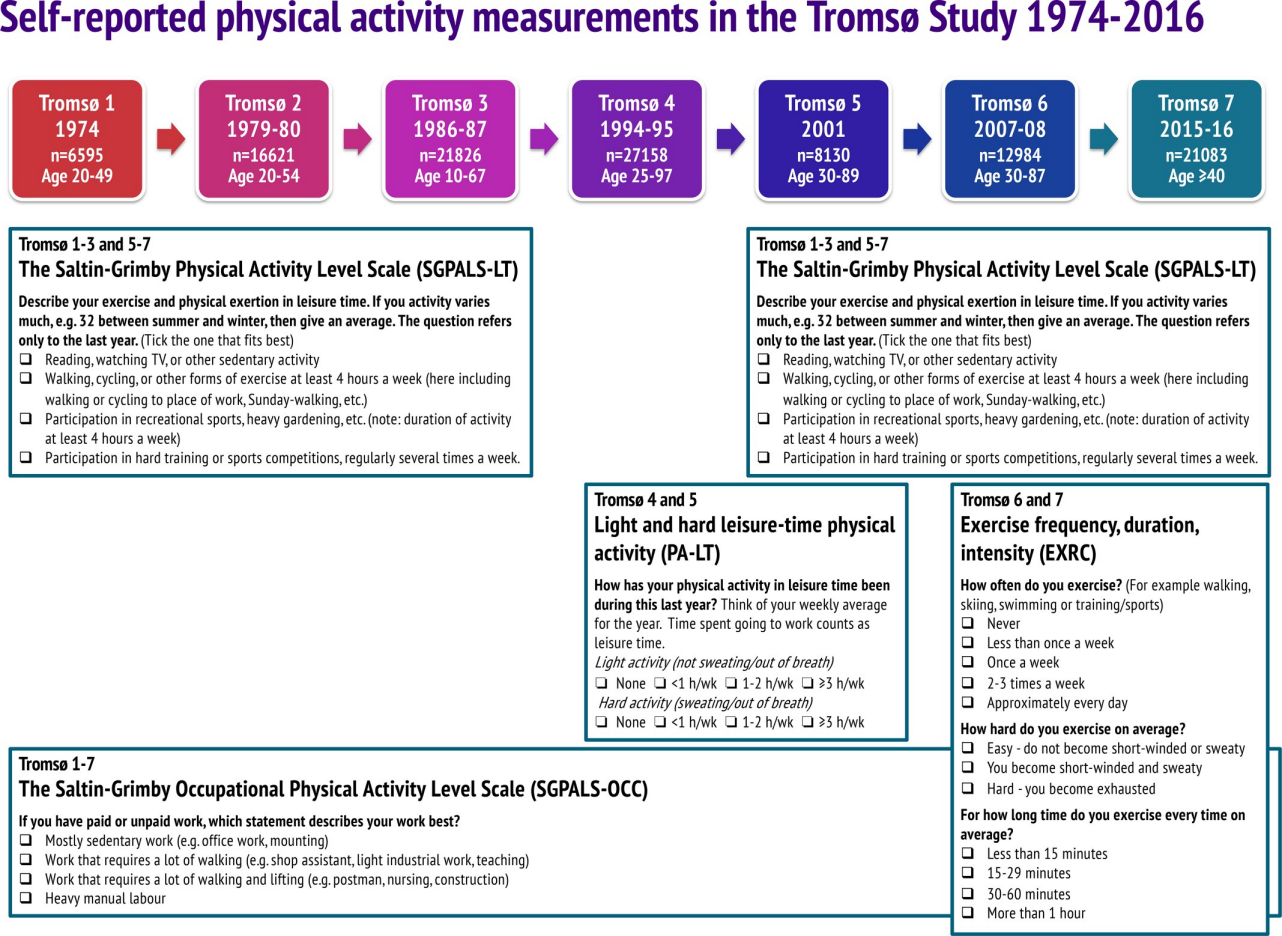
Self-reported measurement of physical activity in the Tromsø Study 1974–2016 (Tromsø 1–7). Numbers refer to participants with valid data on self-reported physical activity.
